# Comprehensive Analysis of Yoghurt Made with the Addition of Yellow Mealworm Powder (*Tenebrio molitor*)

**DOI:** 10.3390/foods13152416

**Published:** 2024-07-30

**Authors:** Brygida Andrzejczyk, Adriana Łobacz, Justyna Ziajka, Anna Lis, Monika Małkowska-Kowalczyk

**Affiliations:** Department of Dairy Science and Quality Management, University of Warmia and Mazury in Olsztyn, 7 Oczapowskiego Str., 10-719 Olsztyn, Poland; 156581@student.uwm.edu.pl (B.A.); justyna.ziajka@uwm.edu.pl (J.Z.); anna.lis@uwm.edu.pl (A.L.); monika.malkowska@uwm.edu.pl (M.M.-K.)

**Keywords:** *Tenebrio molitor*, edible insects, yoghurt with insects, mealworm, nutritional enrichment

## Abstract

The study aimed to be able to incorporate mealworm powder into yoghurts to increase protein content. *Tenebrio molitor* is gaining popularity as an alternative protein source. In the face of a growing human population and the associated challenges of sustainable nutrition, mealworms offer a promising solution. The mealworm is distinguished by its high protein content and for being rich in essential amino acids, vitamins, and minerals, making it a valuable component of diet. Among others, chemical composition, colour, texture, degree of syneresis, sensory analysis, and microbiological analysis were analysed. As expected, the addition of insect powder increased the protein content from 4.91% (0 TM) to 7.41% (5 TM). It also increased the fat content from 1.67% (0 TM) to 3.88% (5 TM). Furthermore, the addition of mealworm powder resulted in a significant change in the colour of the yoghurt to a darker colour, due to the dark brown colour of the powder. Food with added edible insects is increasingly available but is not always popular due to cultural and psychological barriers. Sensory analysis of yoghurts has shown that the more powder that is added, the less appealing the product becomes and the more bitter the taste.

## 1. Introduction

Over the past few years, the world has been facing several challenges, of which the issue of food is becoming the highest priority due to the search for alternative sources of food, as well as protein. According to data from the Food and Agriculture Organisation of the United Nations (FAO), it is estimated that by 2050 the population will be up to 9 billion, and this will involve ensuring adequate food for all [1]. Standard livestock production providing humans mainly with protein containing essential amino acids may not be sufficient. The rearing of pigs, poultry, and cattle requires substantial amounts of energy and space and large quantities of water and feed. Additionally, there are environmental concerns, such as increased greenhouse gas emissions [2,3].

One solution could be to use edible insects as an alternative source of protein exhibiting high nutrient value along with potentially health-promoting components. They are rich in protein and lipids and contain a significant amount of fibre in the form of insoluble chitin. In addition to macronutrients, some insect species are distinguished by their richness in minerals and vitamins [4]. *Tenebrio molitor* larvae (mealworms) have emerged as an alternative and sustainable food sources, as they are a rich source of nutrients (rich protein content, balanced amino acid profile, fatty acids with health benefits, vitamins, and minerals). Meal-worms have received approval for safe human consumption by the European Parlia-ment and Council Authorising the Placing on the Market of Dried Tenebrio molitor Larva as a Novel Food [5].

Yellow mealworm (*Tenebrio molitor*) is generally considered a good source of nutrients. The protein content of the larvae ranges from 41 to 66% (on a dry matter basis) and are considered to be of high quality and with high persistence potential compared to other sources. The amino acid profile of yellow mealworms is rich in leucine, isoleucine, lysine, tyrosine, valine, and methionine, which covers the need for essential amino acids. Insects with a high protein content such as yellow mealworms have a lower energy content and per 100 g provide approximately 206 kcal [6].

Fat is the second most abundant component in the tissues of *Tenebrio molitor.* The lipid content of the larvae varies from 15 to 50% *w*/*w* (on a dry weight basis). They are rich in monounsaturated fatty acids (MUFA), saturated fatty acids (SFA), and polyunsaturated fatty acids (PUFA). The amount of SFA ranges from 23 to 34%, with the two main components being palmitic (C16:0) and stearic (C18:0) acids. The monounsaturated acid (MUFA) content ranges from 37 to 55% and includes palmitoleic acid (C16:1) and oleic acid (C18:1), while the PUFA content (11.28–39.85%) comprises mainly linoleic acid (C18:2) [7].

Consuming insects cannot pose any hazard to consumers’ health and lives. Both naturally occurring insects and those raised in special farms may contain dangerous microorganisms such as pathogenic bacteria, viruses, fungi, and others. However, these pathogens usually present in insects are taxonomically different from those found in vertebrates, which means that they can be considered safe for humans. Furthermore, the bacterial flora of their gut does not pose a threat to human health [8].

Despite being considered a good food source, insects still cause reluctance to be eaten because they are considered generally unattractive. A study undertaken by Tan et al. (2015) shows that the way such products are prepared significantly affects their sensory acceptance among consumers who traditionally do not consume insects [9]. Insects served in a form reminiscent of familiar products or used as a processed ingredient added to traditional products favourably affect the organoleptic experience. First impressions of a product are the most important, as humans judge appearance at the outset. Therefore, insects designated for food production are commonly used in the form of powder, flour, and paste, or their protein is extracted to make it invisible and convince the consumer [8]. Increasingly, products such as pastries, snacks [10,11,12], bread [13], and bars [14,15] are enriched with edible insects such as *Tenebrio molitor*, affecting not only the nutritional value but also the technological, sensory, and final characteristics of the product. The range of insect-based products available on the market is growing systematically. However, due to the new food category and certain production and sales regulations, the availability of products with added insects may be limited between countries. Monitoring the availability of mealworm products in physical and online retail outlets reveals that only whole mealworms are currently available for purchase. However, ongoing research is investigating the incorporation of mealworms into various food products, including bakery items and ice cream [16].

The primary objective of this study was to investigate the feasibility of incorporating mealworm powder into yoghurt formulations to enhance their protein content. Additionally, the study aimed to analyse the physicochemical properties and nutritional composition, conduct microbiological assessments, and perform sensory evaluations of the resultant products.

## 2. Materials and Methods

### 2.1. Insect Processing and Yoghurt Production

The material for the study was yoghurts produced at the Department of Dairy Science and Quality Management, Faculty of Food Science, University Warmia and Mazury in Olsztyn. Four variants of yoghurts were produced with different contents of insect powder additive, as according to Table 1.

Yoghurts were produced using the thermostatic method. The raw materials used were pasteurised milk (Mlekovita, Wysokie Mazowieckie, Poland) with 2% fat content and skimmed milk powder (Spółdzielnia Mleczarska w Gostyniu, Gostyń, Poland). The milk was inoculated with starter cultures YC-X11 of *Lactobacillus delbrueckii subspecies bulgaricus* and *Streptococcus thermophilus* (Chr. Hansen, Horsholm, Poland). Insect powder was produced by freezing dried yellow mealworm larvae (Ovad, Olsztyn, Poland) at −88 °C for 20 h and then grinding in the Speedcook, making 1200 rotations per 1 min.

Yoghurt production started with heating the milk to 42 °C, followed by the addition of the insect powder, skimmed milk powder (in the defined proportions), and yoghurt bacterial culture. The next stage of yoghurt production was the acidification to pH 4.6 at 42 °C, which was monitored using a multi-channel pH/pC/mV multiplexer (Cerko, Gdynia, Poland) equipped with ERH-13-6-type combination electrodes (HYDROMET, Gliwice, Poland). The acidification process required approximately 6 h for all yoghurt variants. After reaching pH 4.6, the yoghurts were rapidly cooled to 4 °C. The obtained yoghurts were stored for 21 days at 4 °C.

### 2.2. Nutrient Composition

The chemical composition of the yoghurts was analysed using a FoodScanTMLab analyzer (FOSS, Poland). This instrument allows the identification of components such as fat, protein, and dry matter in the test material. A yoghurt sample was placed in a petri dish and then introduced into the measuring cuvette of the instrument. The method of analysis was based on near-infrared transmission in the 850–1100 nm range (NIRS). A specially developed computer program calculated the average content and presented the percentage of each component in the yoghurt sample, covering protein, fat, and dry matter.

### 2.3. Acidity

The pH was measured using the Elmetron pHmeter model CP-411 (Zabrze, Poland), which was calibrated before the measurement.

A 0.25 M NaOH solution was utilised with a phenolphthalein indicator to titrate the milk and yoghurt samples (25 g yoghurt + 25 g water). The titration was carried out until a faint pink colouration, persisting for approximately 30 s, was observed. This measurement was performed in duplicate, following the guidelines of the Polish standard.

### 2.4. Syneresis of Whey

Whey syneresis was investigated using two methods. For the first method, the Thermo Scientific Heraeus Megafuge 16 centrifuge was used. A yoghurt sample (±20.5 g) was weighed into a 50 mL tube and then centrifuged (at 10 °C for 15 min at an acceleration of 3300 g). At the end of the centrifugation, the separated whey was weighed, and its percentage in the tube was calculated.

The second method consisted of weighing ±20.5 g of yoghurt and placing it on a sieve. The whey was run at ambient temperature for 2 h. The results in both methods were given as percentages of whey separated from the yoghurt sample.

### 2.5. Assessment of Viscosity

The samples were analysed by performing an oscillation time hardening test using a Physica MCR 102 rheometer (Anton Paar GmbH, Graz, Austria) controlled by RheoCompass software (version 1.31.69, Anton Paar, Graz, Austria). Each sample (20 mL) was poured into the concentric cylinders (CC27) of the rheometer and tested at a temperature of 42.0 ± 0.1 °C using constant values of strain (1%) and frequency (1 Hz). The values of elastic modulus (G′) and viscosity (G″) were recorded continuously to measure changes in the viscoelastic behaviour of the samples during coagulation.

### 2.6. Texture of Yoghurts

Using a TA.XT Texture Analyser (Stable Micro Systems Ltd., Godalming, UK) with Texture Exponent 32 software, texture characteristics were measured. The texture of the yoghurts was determined using a penetration test with an aluminium cylindrical probe to a depth of 20 mm (50% of the original height) and with a contact force of 0.098 N and a speed of 1.0 mm/s. Yoghurt penetration was carried out in 50 cm^3^ plastic cups. Yoghurt samples in unit containers were stored at 4 ± 1 °C. The obtained curves were used to determine firmness (expressed as a maximum penetration force [N]), consistency [N∙s] (expressed as the area of the curve measured to the point of maximum force (firmness) (range of recorded positive loads)), cohesiveness [N] (expressed as the maximum force during probe return, which is taken as an indicator of sample cohesion—the more negative the value, the more ‘cohesive’ the sample is (range of negative loads recorded)), and index of viscosity [N∙s] (expressed as the area of the negative area of the curve—the higher the value, the more resistant the sample is to pullout, indicating greater sample viscosity).

### 2.7. Assessment of Colour

A CM-3500d spectrophotometer (Konica Minolta, Tokyo, Japan) was used to measure colour. Reflectance spectrophotometry in the visible light range (400 to 700 nm) was used to record the spectrum of the test samples. The reflectance was measured by placing a Petri dish with the test sample on an aperture mask (CM-A122) with a field diameter of 8 mm in such a way that the measurement field of the aperture remained completely covered. The surface of the sample was illuminated with diffuse light at an angle of 8° (d/8) and was then reflected and scattered. A light source was used in the colour measurements: illuminant D65. The analysis was carried out using CM-S100w SpectraMagicTM NX Lite Version 2.3 software. The instrument was calibrated with a white (CM A120) and a black (CM A124) standard before the test. The CIE Lab system determined the colour based on the following parameters: L*—colour brightness ranging from 0 (black) to 100 (white), a*—colour-defining parameter positioned as −green/+red, b*—colour-defining parameter positioned as blue/+yellow. Before starting the measurements, the instrument was calibrated on a white standard.

### 2.8. Particle Size

Particle size was measured using a Mastersizer 3000 (Malvern Instruments Ltd., Malvern, UK) with a HydroEV attachment. Deionised water (Millipore Milli-Q, Millipore SAS, Molsheim, France) was used as a dispersant. The yoghurt sample was diluted with deionised water in a ratio of 1:1. Drop by drop, the diluted yoghurt sample was added to the dispersant water until a turbidity value of 14–16% was reached. De-ionised water was used as the continuous phase (refractive index = 1.33), and milk fat was used as the dispersed phase (refractive index = 1.44). The particle sizes of the samples were determined from the measurements:

– dv10—below this value is 10% of the total distribution,
– dv50—median—divides the distribution into 2 equal parts: 50% above and 50% below this value,
– dv90—90% of the distribution lies below this value, and the mean diameters:
– d_43_—de Brouckere’s diameter: d43 = Σn_i_d_i_^4^/Σn_i_d_i_^3^,
– d_32_—Sauter diameter: d_32_ = Σn_i_d_i_^3^/Σn_i_d_i_^2^,

where n_i_—number of particles with diameter d_i_.

### 2.9. Microstructure of Yoghurts

The structure of the yoghurts was analysed using a scanning microscope. Yoghurt samples were observed in their natural state in ESEM mode; this involved carefully applying the sample to a special module designed for use on a Petri table in an FEI model Quanta 200 microscope. Samples in the microscope chamber on the Petri table were frozen to −10 °C and then observed. A Dual BSD detector was used, where:

– Magnification 100×,
– Image size 1.02 mm,
– Electrode acceleration voltage 30.000 kV,
– A scale of 500 μm.

### 2.10. Microbiological Analysis

The total viable count (TVC) was determined using the plate method with surface inoculation on CASO (Merck) microbiological medium (prepared according to the manufacturer’s instructions). Decimal dilutions were made for each variant using 9 mL of sterile peptone water. A 0.1 mL aliquot was inoculated onto three plates and spread evenly on the medium using sterile heads. The plates were incubated under aerobic conditions at 37 °C for 48 h. For counting, plates with a grown number of microorganisms in the range of 30 to 300 were selected. The number of colony-forming units (L) of bacteria was calculated using the following formula:

L = C × d × a [cfu/mL],

where C—sum of colonies on all plates selected for counting, d—dilution factor, and a—factor for the amount of material held.

### 2.11. Sensory Analysis

Organoleptic sensory evaluation of produced yoghurts was carried out at the Department of Dairy Science and Quality Management in Olsztyn using the profile method. The team of assessors consisted of 10 people associated with dairy technology. Each evaluator was given pre-prepared samples and an evaluation sheet.

### 2.12. Statistical Analysis

The mean and standard error (±SEM) for the yoghurt sample results were calculated using Statistica software (version 13.1, 1984–2016, StatSoft, Inc., Tulsa, OK, USA). An analysis of variance (ANOVA) and Tukey’s test (*p* < 0.05) were conducted for all sample determinations, including colour, texture, and syneresis of the yoghurt.

## 3. Results and Discussion

### 3.1. Nutrient Composition

The protein and fat content of the yellow mealworm powder, according to the manufacturer (Ovad, Poland), were 49.9 and 32.4%, respectively. Due to its high protein content, the insect powder was used in the production of yoghurt. Table 2 shows the chemical composition of the yoghurts produced in the present study.

The obtained results showed that the protein content of the yoghurts ranged from 4.59 to 7.41%. The yoghurts with 5% insect powder content were the richest in protein, indicating that insects are an alternative way to increase protein. The sample without added insect powder contained the least protein, where the amount was 1.5 less than the sample with 5% mealworm powder.

The fat content of the produced yoghurts ranged from 1.67% to 3.88%. The yoghurt without added insect powder had the lowest fat content at 1.67%, whereas the sample with 5% added insect powder had the highest fat content at 3.88%. Thus, it can be concluded that insects are also rich in fat, as the higher the addition of insect powder, the higher the fat content of the produced yoghurt. Rumpold and Schlüter (2013) [17] also found that edible insects are rich in nutrients such as protein, fat, vitamins, and minerals. The dry matter content of the resulting yoghurts ranged from 15.3% to 18.77%, with an increase in dry matter, similar to the increases in protein and fat content, observed with greater additions of insect powder. All yoghurts with insect powder addition had a lower water content compared to the control yoghurt (without insect addition), which may indicate a lower water absorption capacity by the insect powder, which affected the properties of the yoghurts.

The dry matter content of the yoghurts varied due to the different ingredient content. The lowest dry matter (e.g., 15.3%) content was found in the control yoghurt (without added insect powder), while the highest dry matter content was obtained in the case of the yoghurt with 5% added mealworm powder.

### 3.2. Acidity Profile during Fermentation

**Figure 1:**
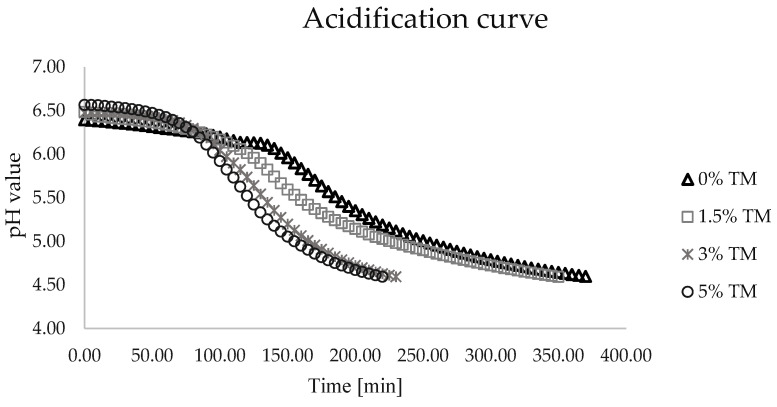
Acidification curves obtained during yoghurt production. 0% TM—control yoghurt without insect powder; 1.5% TM—yoghurt with 1.5% insect powder; 3% TM—yoghurt with 3% insect powder; 5% TM—yoghurt with 5% insect powder.

The correct course of acidification of fermented products is an important step that shapes the attainment of the right organoleptic properties by obtaining the correct acidity and stability of the curd [18]. The course of pH value changes in all yoghurts is shown in Figure 1.

After the addition of the activated yoghurt culture, the initial pH values were between 6.39 and 6.57 (Figure 1). As the fermentation time progressed, the acidity of the yoghurts increased.

The yoghurt containing 5% insect powder reached its isoelectric point at pH 4.6 in 3 h and 40 min, while the yoghurt with 3% insect powder achieved the same pH in 3 h and 50 min. The pH value of 4.6, marking the completion of the fermentation process, was reached by the yoghurt with 1.5% insect powder after 5 h and 50 min. In contrast, the fermentation process of the control yoghurt (0% insect powder) took 20 min longer.

Thus, by observing the results of the fermentation course, it can be concluded that the fermentation time was influenced by the addition of insect powder and the varying lactose content. The greater the addition of mealworm powder, the faster the fermentation process. On the other hand, the prolongation of the fermentation process was due to the content of simple sugars formed during the breakdown of lactose in the fermentation medium.

### 3.3. Profile of Yoghurts’ Acidity during Storage

The results regarding the pH values of the yoghurts during storage are shown in Table 3. 

Upon reaching a pH of 4.6 during fermentation, indicating the completion of the process, the yoghurts were immediately cooled and stored at 4 °C. Over 21 days of storage, there was an average decrease in acidity by 0.23 pH units for each yoghurt type, demonstrating appropriate activity of the yoghurt starter culture used.

The acidity of natural yoghurt corresponding to the preferences of most consumers is 4.2–4.5 pH [19]. On day 21, the pH values of all yogurt variants ranged from 4.29 to 4.41, remaining within the specified range. The potential acidity expressed in °SH is shown in Table 4.

An increase in titratable acidity was observed across all yogurt variants. According to the Polish standard, the titratable acidity for yoghurt should range between 35 and 38 °SH. For the control yoghurt (0% insect powder), the initial titratable acidity was 48 °SH, and it increased by 8.6 °SH after 21 days. The yoghurt with the highest addition of insect powder exhibited the greatest increase in titratable acidity, with a difference of 12.2 °SH. This was followed by the sample with 3% insect powder, which showed a difference of 9 °SH. The sample with 1.5% insect powder addition displayed the smallest change in titratable acidity between days 1 and 21.

An increase in titratable acidity and a decrease in pH is a natural phenomenon for fermented products resulting from the acidifying activity of the yoghurt bacteria. Low storage temperature contributes to the inhibition of vital processes but does not inhibit them completely [20].

### 3.4. Syneresis of Whey

Whey syneresis in yoghurts was measured using two methods: the centrifuge method (Table 5a) and the sieve method (Table 5b).

Comparing the results presented in Table 5a,b, significant differences can be seen between the centrifuge and sieve methods. The centrifuge method is more precise and gives more accurate results. From the data presented in Table 5a, it can be seen that whey syneresis increases significantly with storage time and with the greater addition of mealworm powder. Whey syneresis occurs during the storage of yoghurt and is a serious defect that can lead to the rejection of the product by the consumer [21]. Syneresis during yoghurt storage is associated with severe casein network rearrangements that promote whey leakage [22]. Observing the results after 1 day of production, a significant increase in syneresis was noticed for yoghurts with a higher addition of mealworm powder. The syneresis in the control yoghurt (without added insect powder) was statistically different (*p* < 0.05), with an 11% lower degree of syneresis than the syneresis in the yoghurt with the highest addition of insect powder (5%). The level of syneresis in the control yoghurt was the lowest. This is due to the highest addition of skimmed milk powder, which reduces or prevents syneresis by reinforcing the proteins with dry milk components [23].

The largest differences between samples were observed after 21 days of production, specifically between the control yoghurt (0% insect powder) and the yoghurt containing 5% insect powder (*p* < 0.05). Consequently, it can be concluded that the addition of mealworm powder does not enhance the water retention capacity. The degree of syneresis was also assessed using the sieve method, but this method proved to be less accurate, and the results were not consistent with those obtained from the centrifuge method (Table 5a,b).

Various techniques exist to minimise whey syneresis in set yogurt, including increasing the overall solids content of the milk, subjecting the milk to high-temperature processing, enhancing the pressure during homogenisation, or incorporating stabilising agents such as gelatin, pectin, starches, or gums, which can interact with the casein structure. Several natural ingredients have been identified with unique properties that improve the sensory quality and consistency of set yogurt. A study by Junjun et al. (2023) [23] demonstrated that the incorporation of linoleic acid (LA) decreased whey release, with a positive correlation between the amount of LA added and the reduction in whey syneresis. This effect was attributed to LA’s ability to promote the cross-linking of milk proteins, resulting in a more compact and uniform microstructure in the set yogurt. Consequently, LA not only inhibits whey syneresis but also increases the content of functional fatty acids, significantly enhancing the quality of yogurt products [24].

### 3.5. Texture of Yoghurts

Table 6 shows the texture of the yoghurts tested for 1, 7, 14, and 21 days.

The texture of yoghurts depends on many factors such as dry matter content, composition, temperature and time of initial heat treatment, mechanical treatment of the coagulum, use of stabilisers, type and amount of starter culture introduced into the fermentation, homogenisation process, acidity and heat treatment of the milk, fermentation temperature, and storage conditions of the final product [25].

The hardness of yoghurt is defined as the ability of the specimen to resist deformation before external forces are applied, whereas constancy is the strength of the internal bonding that makes up the bulk of the product [20].

Based on the tests carried out in the present study of yoghurt with added insect powder, it was found that firmness and consistency decreased with increasing levels of added mealworm powder and with the storage time of the yoghurts, while cohesion and viscosity index increased (Table 6). This is related to the altered ratio of mealworm powder and skimmed milk powder. The structure is based on casein chains or clusters of casein micelles interacting with each other and with denatured whey proteins trapped in serum and fat globules. To obtain good rheological properties in yoghurts, the industry typically uses fat-free milk supplementation and heat treatment to denature whey proteins to allow better interaction with casein proteins [26].

### 3.6. Assessment of Colour Parameters

The results of the analysis of colour parameters of the produced yoghurts in terms of L*, a*, and b* values can be found in Table 7.

Increasing the content of mealworm powder resulted in a decrease in the brightness (L*) and an increase in the redness (a*) and yellowness (b*) of the yoghurt samples. A significant difference in the total colour difference was observed between the produced yoghurt samples. As the amount of mealworm powder increased, the yogurts exhibited darker colouration. Similar findings were reported by Zielińska (2023) [27] in studies on ice creams enriched with mealworm powder, where an increase in mealworm powder also led to decreased L* values and increased a* and b* values.

Similar studies with mealworms have been conducted for muffins cakes and bread [28,29]. In baked products, colour changes may be exacerbated by Maillard reactions, which are associated with the increased protein content and its transformation during heat treatment. In products such as yoghurt, the colour change is primarily attributable to the properties of the added ingredients.

### 3.7. Particle Size of Produced Yoghurts

**Figure 2:**
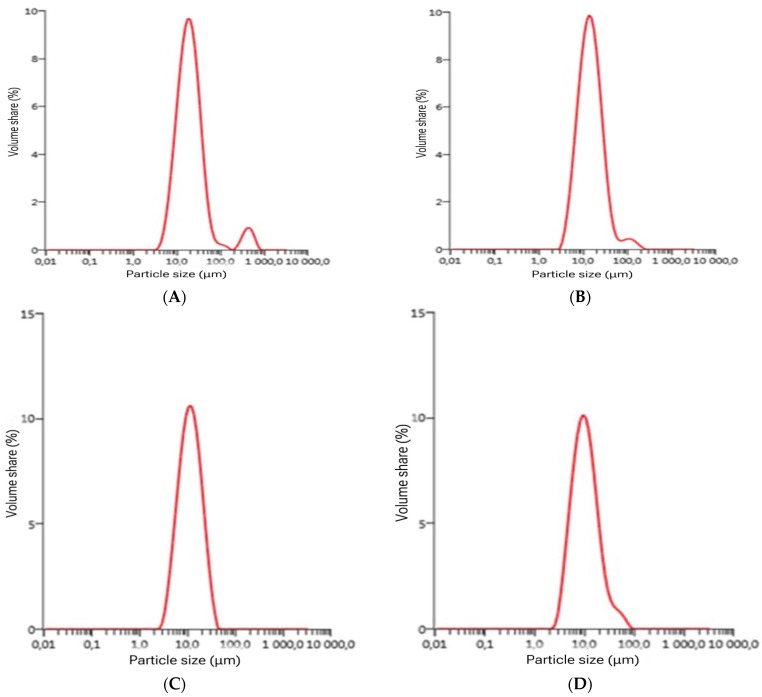
Particle size of produced yoghurt: (**A**)—0 TM: control yoghurt without insect powder, (**B**)—1.5 TM: yoghurt with 1.5% insect powder, (**C**)—3 TM: yoghurt with 3% insect powder, and (**D**)—5 TM: yoghurt with 5% insect powder.

The effect of mealworm powder addition on particle size was carried out after 1 day of production and is shown in Figure 2.

Particle size influenced yoghurt texture, particularly creaminess, and particle size in gels above 150 μm hinders the perception of yoghurt creaminess [30]. Figure 2 shows that the particle size distributions in the samples were similar to each other. The particle sizes in all variants of produced yoghurt had an oblique monodisperse pattern. Yoghurt 3 TM (3% insect addition) showed a monodisperse distribution of particles with the largest percentage of particles >10 μm in diameter; a similar distribution was noted in yoghurts 5 TM and 1.5 TM with a slightly lower percentage of particles. The percentage of particles in the control yoghurt (0 TM) was in the range of 10%, and the largest proportion was >10 μm diameter particles with a smaller proportion of >100 μm diameter particles.

A study conducted by Brauss et al. (1999) [31] shows that particle size decreases with increasing fat content, which was confirmed also in the present study in the case of yoghurts with 3 and 5% mealworm powder additions.

### 3.8. Assessment of Viscosity

Table 8 shows data on the clot formation process during production of all variants of yoghurt (0, 1.5, 3, and 5% insect powder addition).

Yoghurt is a viscoelastic material with rheological properties that can be described by the storage modulus (G′), which determines its degree of elasticity, and the loss modulus (G″), which is a measure of its viscosity [32]. The moment of clot formation is determined when the loss factor is equal to 1 (tanδ = G″/G′ = 1) [33]. The longest clot formation for the control yoghurt (0 TM) was 158 min, and for the yoghurt with the addition of 1.5% mealworm powder (1.5 TM), it was 138 min. The larger addition of mealworm powder resulted in a shorter time for curd formation. The yoghurt with 3% mealworm powder had the shortest curdling time, 16 min, while in the yoghurt with 5% mealworm powder, the curdling time was 24 min.

Table 9 shows data on the viscoelastic properties of produced yoghurts (control and with insect powder addition on 3 levels).

If the storage modulus (G′) is greater than the loss modulus (G‴), then the product retains the viscoelastic properties of a solid (G′ > G″). The opposite is true for a viscoelastic liquid (G′ < G″). From the data presented in Table 9, it can be concluded that all yoghurt variants exhibited viscoelastic solid properties (G′ > G″). The highest loss factor value for a time of 175 min was observed for yoghurt 0 TM and was 0.4491 [−]. The loss factor values decreased with increasing addition of miller powder. For yoghurt 5 TM, the loss factor value was more than 2 times lower and was 0.2065 [−]. 

### 3.9. Microstructure of Yoghurts

**Figure 3:**
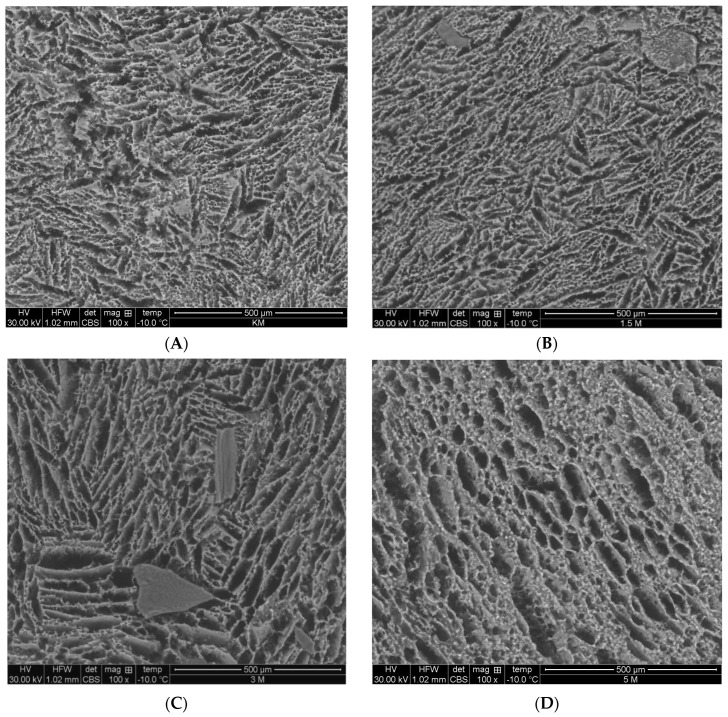
Microstructure of produced yoghurts: (**A**)—0 TM: control yoghurt without insect powder; (**B**)—1.5 TM: yoghurt with 1.5% insect powder; (**C**)—3 TM: yoghurt with 3% insect powder; (**D**)—5 TM: yoghurt with 5% insect powder.

The structure of produced yoghurts was examined by electron microscopy (SEM). Figure 3 shows the structure of all yoghurts determined by SEM (A, B, C, D).

The addition of mealworm powder increased the protein and dry matter values, an increase which resulted in the formation of a gel network by chopping during yoghurt production. The SEM images differed because the addition of the mealworm powder affected the microstructure of yoghurts. The biggest difference was observed between the structures of the 0 TM and 5 TM yoghurts.

The microstructures of yoghurts 0 TM and 1.5 TM are more compact than the others, and the pore size in the gel network of yoghurt 0 TM is significantly smaller than those of yoghurts 3 TM and 5 TM. It was observed for yoghurt with 5% insect powder addition that by simultaneously obtaining the largest pores, a higher level of whey syneresis occurred. According to Vasilean and Segal (2011), the lower amount of dry matter makes it possible to obtain a gel matrix with a large capillary, sufficient to inhibit and avoid whey syneresis [34].

All yoghurt variants were characterised by individualised casein fibres, the smallest being found in yoghurts with 5% insect powder addition. According to Barakat et al. (2021), the individualised casein fibres are due to interactions between denatured caseins and β-lactoglobulin [35].

In Figure 3B,C, irregular large particles were observed, likely resulting from the addition of insect powder with insufficient fineness.

### 3.10. Microbiological Analysis

The counts of the total viable count in the yoghurts during storage are shown in Table 10.

In all yoghurt variants, an increase in the number of microorganisms was observed between 1, 7, 14, and 21 days. On day 1, the lowest number of microorganisms was observed in the control yoghurt (8.17 log cfu/mL), whereas the highest level of bacteria was found in the yoghurt with 5% insect powder addition (8.39 log cfu/mL). An analogous increase in the number of microorganisms was also observed on days 7, 14, and 21.

### 3.11. Sensory Analysis of Yoghurts

Sensory evaluation was carried out over 21 days. To carry out the sensory evaluation, the samples were standardised, so characteristics such as delamination and whey leakage could not be assessed. The results of the yoghurt assessments during storage are shown in Table 11.

Considering the overall evaluation carried out over 3 weeks, the 0 TM yoghurt (0% insect powder addition) scored best, and the yoghurt with 5% mealworm powder scored weakest, in the same way the evaluators rated the attractiveness of the products. When assessing the colour of the products after 1 day of production, the 0 TM yoghurt performed best, while the sample with the addition of 5% mealworm powder addition performed best after 21 days of storage.

The sour and yoghurt-like aroma was most pronounced in the 0% insect powder yoghurt, with its rating decreasing as the proportion of insect powder in the sample increased. Similarly, the sour and yoghurt flavours were assessed in the same manner. Conversely, extraneous taste and odour, as well as bitterness, were rated inversely; these descriptors became more noticeable with higher additions of insect powder.

During the sensory evaluation carried out over 21 days, the smoothest texture was recorded for the 0 TM yoghurt variant. The texture of all yoghurts was not rated as lumpy by the evaluation group. In contrast, the variants with 3% and 5% additions of insect powder were assessed as slightly sandy.

The higher the addition of insect powder, the smoother the consistency of the yoghurts, while the malleability of the yoghurts decreased.

## 4. Conclusions

This research was undertaken to identify the potential of edible insects as a nutritional enhancement in yoghurt production. Edible insects can be incorporated into traditional products mainly to increase protein content. This study showed that with increased levels of mealworm powder, the protein content increases, as well as the fat content (5 TM). The increased level of mealworm had a significant effect on the physico-chemical, microbiological, and sensory analysis of the final product. The yoghurt with the highest addition of mealworm powder (5 TM) had the highest degree of whey syneresis, which could be perceived as much of a disadvantage by the consumer. The dark colour of the yoghurt was due to the increasing insect powder content and the dark brown colour. Sensory analysis, on the other hand, showed that the greater the addition of mealworm powder, the less appealing the product was to the consumer through its bitter taste. A solution to this could be to combine insect-based yoghurt with a fruit additive to modify the sensory properties of the final product. Therefore, research on the incorporation of insects into food should be continuously conducted and refined to gather more information.

## Figures and Tables

**Table 1:** Addition [%] of mealworm powder and skimmed milk powder to yoghurts.

Ingredient	0 TM	1.5 TM	3 TM	5 TM
Mealworm powder	0	1.5	3	5
Skimmed milk powder	5	3	1.5	0

0 TM—control yoghurt without insect powder; 1.5 TM—yoghurt with 1.5% insect powder; 3 TM—yoghurt with 3% insect powder; 5 TM—yoghurt with 5% insect powder.

**Table 2:** Nutritional composition (mean ± standard deviation) of yoghurts.

	0 TM	1.5 TM	3 TM	5 TM
Fat (%)	1.67 ± 0.38	1.75 ± 0.31	2.58 ± 0.32	3.88 ± 0.52
Protein (%)	4.91 ± 0.13	5.26 ± 0.22	7.22 ± 0.45	7.41 ± 0.43
Moisture (%)	84.6 ± 0.13	84.55 ± 0.62	82.35 ± 0.18	81.44 ± 0.10
Dry matter (%)	15.3 ± 0.15	15.35 ± 0.62	17.76 ± 0.18	18.77 ± 0.13

0 TM—control yoghurt without insect powder; 1.5 TM—yoghurt with 1.5% insect powder; 3 TM—yoghurt with 3% insect powder; 5 TM—yoghurt with 5% insect powder.

**Table 3:** Changes (mean ± standard deviation) in pH values of produced yoghurts during storage.

Day	0 TM	1.5 TM	3 TM	5 TM
1	4.6 ± 0.01	4.6 ± 0.01	4.6 ± 0.01	4.6 ± 0.01
7	4.52 ± 0.01	4.38 ± 0.01	4.54 ± 0.01	4.58 ± 0.01
14	4.37 ± 0.01	4.25 ± 0.01	4.31 ± 0.01	4.43 ± 0.01
21	4.39 ± 0.01	4.29 ± 0.01	4.37 ± 0.01	4.41 ± 0.01

0 TM—control yoghurt without insect powder; 1.5 TM—yoghurt with 1.5% insect powder; 3 TM—yoghurt with 3% insect powder; 5 TM—yoghurt with 5% insect powder.

**Table 4:** Changes (mean ± standard deviation) in potential acidity (°SH) of produced yoghurts during storage.

Day	0 TM	1.5 TM	3TM	5 TM
1	48 ± 0.01	50.2 ± 0.01	39.4 ± 0.01	37.2 ± 0.01
7	54 ± 0.01	53.2 ± 0.01	41.8 ± 0.01	40.2 ± 0.01
14	54 ± 0.01	53.4 ± 0.01	46.2 ± 0.01	45.6 ± 0.01
21	56.6 ± 0.01	54.6 ± 0.01	48.4 ± 0.01	49.4 ± 0.01

0 TM—control yoghurt without insect powder; 1.5 TM—yoghurt with 1.5% insect powder; 3 TM—yoghurt with 3% insect powder; 5 TM—yoghurt with 5% insect powder.

**Table 5:** (a) Whey syneresis (% mean ± standard deviation) of yoghurts—the centrifuge method. (b) Whey syneresis (% mean ± standard deviation) of yoghurts—the sieve method.

Day	0 TM	1.5 TM	3 TM	5 TM
(**a**)				
1	21.17 ^bx^ ± 0.42	25.21 ^dy^ ± 1.41	23.37 ^cx^ ± 1.31	32.92 ^bw^ ± 0.73
7	26.23 ^bx^ ± 1.06	29.73 ^cx^ ± 0.17	34.61 ^bw^ ± 0.62	31.99 ^bwx^ ± 0.55
14	39.11 ^ax^ ± 0.99	37.71 ^bx^ ± 1.07	48.57 ^aw^ ± 0.76	48.05 ^aw^ ± 0.18
21	37.19 ^ax^ ± 0.00	48.82 ^aw^ ± 0.92	48.49 ^aw^ ± 0.97	49.45 ^aw^ ± 0.99
(**b**)				
1	20.84 ^ax^ ± 0.66	22.90 ^ax^ ± 0.93	21.68 ^bx^± 0.25	32.04 ^aw^ ± 0.53
7	19.38 ^ax^ ± 0.03	17.06 ^cx^ ± 0.82	28.90 ^aw^ ± 0.77	30.94 ^abw^ ± 0.75
14	18.76 ^ax^ ± 0.90	19.64 ^bcx^ ± 0.39	30.55 ^aw^ ± 1.06	31.76 ^aw^ + 1.12
21	17.25 ^ax^ ± 0.70	20.83 ^abwx^ ± 0.89	25.32 ^abwx^ ± 1.15	26.89 ^bw^ ± 0.79

0 TM—control yoghurt without insect powder; 1.5 TM—yoghurt with 1.5% insect powder; 3 TM—yoghurt with 3% insect powder; 5 TM—yoghurt with 5% insect powder. ^w–y^ Average values in the same row marked with different symbols are statistically significantly different (*p* < 0.05). ^a–d^ The mean values in the same column indicated by different symbols are statistically significantly different (*p* < 0.05).

**Table 6:** Texture parameters (mean ± standard deviation) of the produced yoghurts with added insect powder.

	Sample	Day			
		1	7	14	21
Firmness (N)	0 TM	1.73 ^aw^ ± 0.01	1.71 ^aw^ ± 0.02	1.68 ^aw^ ± 0.02	1.63 ^aw^ ± 0.06
	1.5 TM	1.70 ^aw^ ± 0.05	1.75 ^aw^ ± 0.07	1.60 ^aw^ ± 0.01	1.58 ^aw^ ± 0.10
	3 TM	0.88 ^bw^ ± 0.05	0.89 ^cw^ ± 0.04	0.94 ^cw^ ± 0.06	0.85 ^bw^ ± 0.04
	5 TM	0.66 ^bw^ ± 0.09	0.57 ^bw^ ± 0.07	0.60 ^bw^ ± 0.03	0.62 ^bw^ ± 0.00
Consistency (N·s)	0 TM	25.27 ^aw^ ± 0.21	25.53 ^aw^ ± 0.40	25.16 ^aw^ ± 0.32	24.43 ^aw^ ± 0.48
	1.5 TM	24.79 ^aw^ ± 0.64	26.13 ^aw^ ± 1.47	23.51 ^aw^ ± 0.11	23.44 ^aw^ ± 0.93
	3 TM	11.60 ^bw^ ± 0.91	11.86 ^cw^ ± 0.41	13.08 ^cw^ ± 1.30	11.22 ^cw^ ± 0.16
	5 TM	8.39 ^bw^ ± 1.69	7.15 ^bw^ ± 1.21	8.24 ^bw^ ± 0.72	8.27 ^bw^ ± 0.34
Cohesiveness (N)	0 TM	−1.70 ^dw^ ± 0.07	−1.80 ^cw^ ± 0.08	−1.67 ^cw^ ± 0.02	−1.97 ^bw^ ± 0.28
	1.5 TM	−1.49 ^cw^ ± 0.07	−1.61 ^cw^ ± 0.04	−1.47 ^cw^ ± 0.10	−1.41 ^bw^ ± 0.10
	3 TM	−0.69 ^bw^ ± 0.01	−0.75 ^bw^ ± 0.00	−0.81 ^bw^ ± 0.06	−0.66 ^aw^ ± 0.08
	5 TM	0.45 ^aw^ ± 0.01	−0.40 ^aw^ ± 0.05	−0.39 ^aw^ ± 0.07	−0.40 ^aw^ ± 0.07
Index of Viscosity (N·s)	0 TM	−1.86 ^bw^ ± 0.17	−1.97 ^bw^ ± 0.08	−1.79 ^cw^ ± 0.16	−1.09 ^aw^ ± 0.94
	1.5 TM	−1.71 ^bw^ ± 0.01	−1.65 ^bw^ ± 0.01	−1.70 ^cw^ ± 0.00	−1.84 ^aw^ ± 0.10
	3 TM	−0.75 ^aw^ ± 0.13	−0.75 ^aw^ ± 0.30	−0.92 ^bw^ ± 0.05	−0.79 ^aw^ ± 0.18
	5 TM	−0.46 ^aw^ ± 0.00	−0.38 ^aw^ ± 0.04	−0.39 ^aw^ ± 0.09	−0.44 ^aw^ ± 0.11

0 TM—control yoghurt without insect powder; 1.5 TM—yoghurt with 1.5% insect powder; 3 TM—yoghurt with 3% insect powder; 5 TM—yoghurt with 5% insect powder. ^w^—Average values in the same row marked with different symbols are statistically significantly different (*p* < 0.05). ^a–c^ The mean values in the same column indicated by different symbols are statistically significantly different (*p <* 0.05).

**Table 7:** Colourimetric properties (mean ± standard deviation) of produced yoghurts with addition of insect powder during storage.

	Sample	Day			
		1	7	14	21
L*	0 TM	85.25 ^ax^ ± 0.12	85.24 ^ax^ ± 0.14	85.38 ^ax^ ± 0.13	88.46 ^aw^ ± 0.07
	1.5 TM	81.58 ^bz^ ± 0.36	83.30 ^bx^ ± 0.14	82.42 ^by^ ± 0.17	86.43 ^bz^ ± 0.17
	3 TM	75.90 ^cx^ ± 0.13	69.43 ^cy^ ± 0.72	75.91 ^cx^ ± 0.41	79.07 ^cw^ ± 0.27
	5 TM	67.80 ^dy^ ± 0.82	58.56 ^dz^ ± 0.56	70.41 ^dx^ ± 0.60	76.61 ^dw^ ± 0.67
a*	0 TM	−3.34 ^dx^ ± 0.02	−3.35 ^dx^ ± 0.01	−3.29 ^dw^ ± 0.02	−3.29 ^dw^ ± 0.03
	1.5 TM	−2.62 ^cz^ ± 0.06	−1.83 ^cx^ ± 0.04	−1.63 ^cw^ ± 0.08	−2.19 ^cy^ ± 0.05
	3 TM	−0.55 ^bw^ ± 0.07	−0.27 ^bx^ ± 0.20	−0.11 ^bx^ ± 0.13	0.12 ^by^ ± 0.07
	5 TM	0.14 ^ay^ ± 0.18	1.08 ^ax^ ± 0.16	1.37 ^ax^ ± 0.20	3.39 ^aw^ ± 0.23
b*	0 TM	8.65 ^cy^ ± 0.07	8.96 ^cx^ ± 0.03	8.99 ^cx^ ± 0.05	9.15 ^cw^ ± 0.07
	1.5 TM	8.85 ^cwx^ ± 0.31	9.08 ^cw^ ± 0.11	8.69 ^cx^ ± 0.14	9.09 ^cw^ ± 0.06
	3 TM	10.83 ^bx^ ± 0.15	11.93 ^bw^ ± 0.42	10.95 ^bx^ ± 0.45	10.45 ^bx^ ± 0.40
	5 TM	12.88 ^ax^ ± 0.65	15.35 ^aw^ ± 0.87	12.93 ^ax^ ± 0.81	11.52 ^ay^ ± 0.64

0 TM—control yoghurt without insect powder; 1.5 TM—yoghurt with 1.5% insect powder; 3 TM—yoghurt with 3% insect powder; 5 TM—yoghurt with 5% insect powder. ^w–z^ Average values in the same row marked with different symbols are statistically significantly different (*p* < 0.05). ^a–d^ The mean values in the same column indicated by different symbols are statistically significantly different (*p* < 0.05).

**Table 8:** Average values (±standard deviation) of time, conservation modulus, and loss modulus for each yoghurt variant.

Sample	Time (min)	Conservative Module (Pa)	Loss Modulus (Pa)	Loss Factor (−)
0 TM	158 ± 0.66	0.06428 ± 0.00	0.06428 ± 0.00	1
1.5 TM	137.5 ± 0.71	0.08093 ± 0.02	0.08093 ± 0.02	1
3 TM	16.15 ± 0.57	0.05550 ± 0.01	0.05550 ± 0.01	1
5 TM	24.2 ± 0.00	0.11019 ± 0.00	0.11019 ± 0.00	1

0 TM—control yoghurt without insect powder; 1.5 TM—yoghurt with 1.5% insect powder; 3 TM—yoghurt with 3% insect powder; 5 TM—yoghurt with 5% insect powder.

**Table 9:** Average (±standard deviation) values of the retention and loss modulus for produced yoghurts for a time of 175 min.

Sample	Time (min)	Conservative Module (Pa)	Loss Modulus (Pa)	Loss Factor (−)
0 TM	175	1.81015 ± 0.41	0.8113 ± 0.17	0.4491 ± 0.01
1.5 TM	175	27.0461 ± 0.29	10.6395 ± 0.28	0.3915 ± 0.02
3 TM	175	72.2211 ± 0.95	17.6595 ± 0.24	0.2465 ± 0.04
5 TM	175	76.6524 ± 0.32	15.7565 ± 0.45	0.2065 ± 0.02

0 TM—control yoghurt without insect powder; 1.5 TM—yoghurt with 1.5% insect powder; 3 TM—yoghurt with 3% insect powder; 5 TM—yoghurt with 5% insect powder.

**Table 10:** Total microbial count (log cfu/g ± standard deviation) of produced yoghurts.

Day	0 TM	1.5 TM	3 TM	5 TM
1	8.17 ± 0.67	8.30 ± 0.99	8.38 ± 0.19	8.39 ± 0.68
7	8.19 ± 0.61	8.33 ± 0.14	8.39 ± 0.97	8.40 ± 0.75
14	8.19 ± 0.14	8.33 ± 0.61	8.40 ± 0.15	8.41 ± 0.19
21	8.20 ± 0.07	8.34 ± 0.96	8.41 ± 0.13	8.43 ± 0.32

0 TM—control yoghurt without insect powder; 1.5 TM—yoghurt with 1.5% insect powder; 3 TM—yoghurt with 3% insect powder; 5 TM—yoghurt with 5% insect powder.

**Table 11:** Sensory analysis of yoghurts.

	Characteristic Feature	Average			
		0 TM	1.5 TM	3 TM	5 TM
Appearance	Colour	5.30	4.90	5.10	5.20
	Syneresis	1.70	1.50	2.00	2.80
	Attraction	5.90	5.30	4.30	3.40
	Whey leakage	1.50	1.10	1.10	1.10
Odour	Yoghurt	5.30	4.20	2.90	2.50
	Sour	4.50	3.00	2.70	2.20
	Atypical	1.30	1.70	2.60	3.40
Consistency	Smooth	6.20	4.60	3.80	3.10
	Gross	1.00	1.40	1.40	1.30
	Sandy	1.10	1.80	3.10	3.80
	Fine, Liquid	2.90	3.90	5.70	5.90
	Dense	3.90	3.20	1.90	1.30
	Uniform	6.20	5.80	4.70	4.70
	Ductile	4.80	4.50	3.90	3.50
Taste	Sweet	2.60	2.10	2.10	1.70
	Sour	4.30	3.70	2.90	2.80
	Yoghurt	4.80	3.90	3.00	2.60
	Bitter	2.30	2.30	3.40	3.50
	Atypical	1.60	2.60	4.50	4.90
	General assessment	4.80	4.40	2.44	2.29

## Data Availability

The original contributions presented in the study are included in the article, further inquiries can be directed to the corresponding author.
